# Neuropsychological consequences of drowning in children

**Published:** 2023-12

**Authors:** Sima Garshasbi

**Affiliations:** ^*a* Department of Health in Disasters and Emergencies, School of Public Health, Tehran University of Medical Sciences, Tehran, Iran. ^

## Abstract

**Background:**

Drowning means the entry of water into the lungs and respiratory tract and suffocation as a result of immersion in water. If hypoxia happens, it can damage the neurons in the first few minutes after drowning.

**Methods:**

This is a retrospective analysis in which the studies related to various neurological and psychological outcomes in drowned children were extracted from MEDLINE, Google Scholar, PubMed, and Scopus databases and were reviewed.

**Results:**

The neurocognitive outcomes of drowning cannot be accurately predicted in children in the initial period of treatment. Out-of-hospital and in-hospital therapeutic interventions are necessary. The duration of submersion, the strong management of lifesaving operations at the scene of the accident, the duration of cardiopulmonary resuscitation, and spontaneous breathing and blood circulation when entering the emergency room are the most important factors to be considered. There is scientific evidence that a complete and accurate neurological examination at the time of discharge cannot reveal possible outcomes related to hypoxic brain damage in children. Their neurological examination before discharge can be completely healthy at the time of discharge, but long-term cognitive outcomes can later manifest when children go to school in the future and/or as mental complications in adulthood.

**Conclusions:**

Any sign of drowning in children can lead to serious neurological and psychological outcomes so timely and continuous physical and neuropsychological follow-up of children who experienced drowning is recommended.

**Keywords:**

Neuropsychological, Consequences, Drowned, Child, Outcome, Drowning

